# Effects of minute oscillation stretching training on muscle and tendon stiffness and walking capability in people with type 2 diabetes

**DOI:** 10.1007/s00421-024-05596-y

**Published:** 2024-09-09

**Authors:** Riccardo Magris, Andrea Monte, Francesca Nardello, Michele Trinchi, Nicolò Vigolo, Carlo Negri, Paolo Moghetti, Paola Zamparo

**Affiliations:** 1 Department of Neurosciences, Biomedicine and Movement Sciences, University of Verona, Verona, Italy; 2 Department of Medicine, University of Verona, Verona, Italy; 3 Integrated University Hospital of Verona - Endocrinology, Diabetology and Metabolic Diseases Unit, Verona, Italy

**Keywords:** Muscle mechanics, Tendon stiffness, Diabetes, Training, Stretching

## Abstract

**Aim:**

we investigated the effects of a 10 week training program (i.e., minute oscillatory stretching; MOS) on the mechanical responses and walking capability in people with type 2 diabetes (T2D).

**Methods:**

seventeen T2D patients performed maximum voluntary contractions of the plantar flexor muscles during which Achilles tendon stiffness (k_T_) and muscle–tendon stiffness (k_M_) were evaluated at different percentages of the maximum voluntary force (MVC). In addition, each participant was requested to walk at different walking speeds (i.e. 2, 3, 4, 5, and 6 kmh^−1^) while their net energy cost of walking (C_net_), cumulative EMG activity per distance travelled (CMAPD) and kinematic parameters (step length, step frequency, the ankle/knee range of motion) were evaluated.

**Results:**

maximum tendon elongation increased after MOS training, and k_T_ significantly decreased (between 0 and 20% of MVC). No differences were observed for muscle elongation or k_M_ after training. C_net_ decreased after training (at the slowest tested speeds) while no changes in CMAPD were observed. Step length and ankle ROM during walking increased after training at the slowest tested speeds, while step frequency decreased; no significant effects were observed for knee ROM.

**Conclusion:**

these results indicate the effectiveness of 10 weeks of MOS training in reducing tendon stiffness and the energy cost during walking in people with T2D. This training protocol requires no specific instrumentation, can be easily performed at home, and has a high adherence (92 ± 9%). It could, thus, be useful to mitigate mechanical tendon deterioration and improve physical behaviour in this population.

**Supplementary Information:**

The online version contains supplementary material available at 10.1007/s00421-024-05596-y.

## Introduction

Type 2 diabetes (T2D) is one of the most prevalent chronic diseases in the elderly. The International Diabetes Federation (Sun et al. 2022) estimates that 537 million adults have diabetes worldwide, which may rise to 783 million in 2045 (Sun et al. 2022). In this epidemiological context, diabetes exerts an increasingly significant impact on the healthcare systems. Understanding the molecular, structural, and functional alterations related to diabetes could aid in mitigating its adverse effects and reducing healthcare costs.

Hyperglycaemia in T2D, leads to an increase of advanced glycation end products (AGEs). AGEs are non-enzymatically glycated proteins and lipids created via the formation of Schiff bases, Amadori rearrangement, and the formation of various intermediates in the Maillard reaction. The formation of AGEs is irreversible, and their accumulation can damage several tissues. Soft tissues, such as muscle and tendon, modified by AGEs undergo structural changes due to cross-linkage formation and become stiffer (Burner et al. 2012; Birukov et al. 2021).

In animal models, it was observed that diabetes causes an accumulation of carboxymethyl-lysine (a specific AGE) that leads to muscle dysfunction (Snow and Thompson 2009). In humans, Fang et al. (2024) observed significant differences in muscle stiffness comparing diabetic subjects with healthy controls. Couppé et al. (2016) showed that Achilles tendon stiffness (i.e., the capability of the tendon to resist deformation in response to an applied force) and skin connective tissue cross-linking are greater in diabetic patients and suggested that elevated tendon stiffness may influence gait parameters, increasing the metabolic energy expended for a given distance, i.e. the energy cost of locomotion. Accordingly, Petrovic et al. (2018) observed that diabetic patients display less Achilles tendon elongation, higher tendon stiffness, and higher tendon hysteresis during walking than healthy controls. On the other hand, Caron et al. (2018) and Petrovic et al. (2016) showed higher values of energy cost of walking in people with T2D compared to controls. This is of scientific interest since physical activity is considered a cornerstone for glycaemic control in people with T2D (Colberg et al. 2016) and walking is one of the most important “training strategies” for these patients (Moghetti et al. 2020). Therefore, strategies able to reduce muscle and tendon mechanical alterations imposed by diabetes could help in reducing the functional impact of this pathology.

Physical activity (e.g. training) could improve muscle–tendon function. Indeed, long-term strength and endurance training protocols were observed to lead to improvement of glycated hemoglobin (HbA1c) levels, insulin sensitivity, blood pressure, lipid profiles (Kanaley et al. 2022), as well as muscle strength and physiological output (Bacchi et al. 2012). Strength and endurance training, however, would rather increase (or preserve) tendon stiffness than decrease it (Farris et al. 2012; McCrum et al. 2018); training strategies able to reduce tendon stiffness involve static and dynamic stretching. Indeed, a single session of static or dynamic stretching was observed to decrease muscle–tendon unit stiffness (e.g. Cè et al. 2015; Longo et al. 2014) in healthy subjects, but the decreases in stiffness were accompanied by a temporary decrease in muscle force (Bacurau et al. 2009; Behm et al. 2016). In addition, stretching is known to induce a reduction in maximal torque and in the rate of torque development, which are factors related to functional disability (e.g. risk of falls; Perry et al. 2007). Therefore, static and dynamic stretching training programs are not recommended for people with T2D.

Recently, Ikeda et al. (2019, 2021) proposed a new stretching modality (minute oscillation stretching, MOS) that allows conditioning of the plantar-flexors muscle–tendon units by providing repetitive small longitudinal length changes using a passive stretch of the ankle joint. In young and healthy participants, a single session of unilateral MOS training was sufficient to reduce muscle and tendon stiffness without affecting muscle strength of the tested leg (Ikeda et al. 2019; 2021). Since the plantar-flexor muscles are the most important propulsive muscles for human locomotion (Hamner et al. 2010), and since their muscle–tendon behavior strongly affects the mechanical and physiological responses during locomotion (e.g., walking and running; Monte et al. (2022a, b), Monte et al. (2020)) MOS training can be expected to improve plantar-flexor muscles function, without adverse effects, and thus also locomotor capability in diabetic people.

Hence, in this study, we combined dynamometric, ultrasound, kinematic and metabolic data to evaluate the effects of 10 weeks of oscillatory stretching training on the mechanical responses (Achilles tendon and muscle–tendon unit stiffness) and walking capability (energy cost, EMG activity knee/ankle range of motion) in people with T2D. We hypothesized that: (i) 10 weeks of MOS training will be effective in reducing Achilles tendon and MTU stiffness, increasing tissue elongation without affecting muscle strength; (ii) the decrease in stiffness will be related to an increase in the knee/ankle range of motion during the ground contact phase and to a decrease in the energy cost of walking, due to a higher tendon stretch–shortening capacity after training.

## Materials and methods

### Participants

Twenty-one individuals diagnosed with type 2 diabetes were enrolled in this study. The inclusion criteria included age between 50 and 70 years, BMI between 23 and 30 kg m^−2^, and a moderate level of physical activity (quantified by means of the International Physical Activity Questionnaire, IPAQ, www.ipaq.ki.se). Exclusion criteria included neuropathy, foot ulcers, arterial insufficiency, arthritis of the ankle/foot, previous foot/knee surgery, prior Achilles tendon rupture, prior Charcot foot, cardiovascular and respiratory deficits, and insulin therapy. Besides the IPAQ, other questionnaires were used to determine the level of functional capacity (instrumental activities daily living (IADL) and the activities of daily living (ADL) questionnaires (Lawton and Brody 1969).

The study adhered to the principles outlined in the Declaration of Helsinki, obtained approval from the local ethical committee (CESC AOUI, Verona; protocol number: 40428) and was registered as a clinical trial (ClinicalTrials.gov, protocol number: NCT05585502). After receiving comprehensive information regarding the objectives, methodologies, and potential risks associated with their involvement in the study, all participants provided their informed written consent.

## Intervention

The stretching protocol involved 10 weeks of training, 5 sessions per week (50 sessions in total). The duration of the training protocol was selected in accordance with recent meta-analyses which observed significant differences in tendon mechanical properties after long interventions (≥ 10 weeks) compared to shorter ones (Bohm et al. 2015; Lazarczuk et al. 2022). Each session lasted about 15 min and was composed of five sets of 1 min of oscillatory passive stretching (MOS) at 1 Hz, with a 30 s rest interval between sets (Ikeda et al. 2019, 2021) performed twice: for the right and left lower limb. The participants were requested to sit on a chair and to set their foot over another chair positioned in front of them (the knee angle was, thus, completely extended; see Figure S1 in supplementary materials). The correct oscillatory frequency was given by a metronome and the range of motion was set from maximum dorsiflexion to the normal passive plantarflexion position and was achieved by stretching and releasing a rubber band passed under the sole of the feet. The stretching intensity (self-selected by the participants) was set as the maximal acceptable dorsiflexion angle without severe pain sensation.

The first training session was performed in the biomechanic’s lab, under the supervision of an operator, whereas the following sessions were performed at home (telemedicine training classes); these classes were led by an expert supervisor who monitored attendance, exercise execution and intensity via webcam and a microphone. In particular, the supervisor checked that the range of motion was always around/near the maximal dorsiflexion angle and that the participants avoided any knee bending during the execution of the stretching.

Participants were instructed to record, after each telemedicine session, their perceived exercise intensity through the CR10 scale (from 0 = no effort to 10 = maximal effort; Borg et al. 2010) and any localized ankle pain using a NPRS scale (numeric pain rating scale) (from 0 = no pain to10 = worst imaginable pain; Jensen et al. 1989). Attendance, CR10 and NPRS scores were recorded for each stretching session.

## Experimental design

The outcome measurements related to the muscle–tendon and gait evaluations were obtained at baseline (PRE) and within one week after the last training session (POST).

A preliminary session that included the questionnaire assessment and a blood sampling was performed a few days before the baseline tests. Blood samples of 15 ml were collected according to the standard clinical management of patients with type 2 diabetes to determine serum glucose, triglycerides, HDL cholesterol, total cholesterol, creatinine, and glycated hemoglobin (HbA1c). The 3 year average HbA1c level of each patient was also obtained from the medical records.

Muscle–tendon and gait evaluations were conducted on the same day (in a randomized order) and a minimum rest period of two hours was maintained in between.

In the muscle–tendon evaluation session maximal fixed-end voluntary contractions (MVC) were utilized to investigate medial gastrocnemius (GM) muscle–tendon unit (MTU_GM_) stiffness (k_M_) and Achilles tendon (AT) stiffness (k_T_).

In the gait session, the participants were asked to walk on a treadmill, maintaining a self-selected cadence and step length, at speeds from 2 to 6 kmh^−1^, with 1 kmh^−1^ increments (presented in sequential order). During these experiments, the energy cost of walking, the EMG activity of selected lower limb muscles (vastus lateralis, biceps femoris, tibialis anterior and gastrocnemius medialis), spatiotemporal variables (cadence and stride length) and the range of motion (ROM) of the knee and ankle joints were investigated.

## Data collection

### Muscle–tendon evaluation

A standardized warm-up, based on 30 unloaded calf raises, preceded the muscle–tendon evaluation. Participants were then secured on a dynamometer (Cybex NORM, Lumex Inc., Ronkonkoma, New York, USA) with a trunk and pelvis strap to minimize hip movement; both knee and ankle joints were positioned in the anatomical position (as during the training protocol) and the dominant foot was fixed to the dynamometer footplate. During familiarization, the ankle axis of rotation, defined as the imaginary line connecting medial and lateral malleoli, was carefully aligned to the dynamometer’s axis of rotation during ten sub-maximal voluntary contractions (Arampatzis et al. 2004).

Participants were then instructed to gradually increase force expression (i.e. ankle joint extension) over 2–4 s until reaching their maximum, and to maintain this level of torque for 2–3 s (Maganaris and Paul 2002). Each participant performed at least six MVCs (3 + 3 contractions for the evaluation of MTU and tendon stiffness, respectively) with 2 min of recovery in between (Monte et al. 2021). Visual feedback of the torque profile was provided to optimize force expression; timing and strong verbal encouragement was provided as well. Torque data were sampled at 1000 Hz by means of a PowerLab System (PowerLab, ADInstrument) using the LabChart software (v8.1.13, ADIstrument).

During these experiments, an ultrasound apparatus with a 6-cm linear array probe operating at 60 Hz (Telemed Mycrus Ext-1, Lituania) recorded GM and AT behaviour (e.g., the displacement of the muscle fibres and of the muscle–tendon junction (MTJ), respectively). A 3 V square wave trigger was initiated at the onset of the ultrasound recording to synchronize ultrasound data with the torque signal.

To determine the torque-shortening curve of the MTU_GM_, the probe was placed in the sagittal plane on the most prominent part of the GM muscle belly (Hauraix et al. 2015). For the torque-elongation curve of Achille’s tendon, the same probe was placed along the middle-longitudinal axis of the muscle–tendon unit at the GM muscle–tendon junction (Magnusson et al. 2001). An elastic bandage was used to secure the probe to the skin. To reduce muscle compression, a soft gel pad was interposed between the probe and the skin (Monte 2021). The probe’s position was corrected to achieve a clear visibility of the muscle belly/MTJ and of the surrounding connective tissue (Van Hooren et al. 2020).

### Walking trials

A familiarization period consisting of a minimum of one minute of walking at each tested speed on the treadmill (H/P/Cosmos, Saturn 300/100r, Germany) was proposed to each participant. A wireless EMG system (Aurion, Cometa, Bareggio, Italy) recorded the signals of vastus lateralis (VL), biceps femoris (BF), tibialis anterior (TA) and gastrocnemius medialis (GM) at a sampling frequency of 1000 Hz. Two Ag–AgCl bipolar electrodes were attached to each muscle belly following the SENIAM guidelines (Hermens et al. 2000).

To determine spatiotemporal gait parameters and the range of motion of the knee and ankle joints, a MoCap system (Vicon, Oxford, UK) operating at a sampling frequency of 100 Hz recorded the kinematics of 16 markers placed according to the lower-body PlugInGait marker set. The Nexus software synchronized kinematic and EMG data.

During each walking trial, a breath-by-breath metabolimeter (CPET, Cosmed, Albano Laziale, Italy) was used to assess oxygen uptake (V̇O_2_). At least 10 min after the conclusion of the familiarization phase, baseline data were collected for five minutes in a standing position; V̇O_2_ was then recorded for six minutes at each walking speed. Data collected in the last 60 s of standing or exercise were averaged and used in further analysis.

Kinematics and EMG data were obtained for ten strides during the second and the fourth minute of each testing condition (at each speed).

## Data analysis

The offline analyses were conducted using Matlab (R2022b).

### Muscle–tendon evaluation

Muscle thickness (MT) and pennation angle (PA) of four fascicles of GM were traced frame by frame by means of a customized version of the semi-automatic tracking algorithm developed by Farris and Lichtwark (2016). Following the auto-tracking process, each frame was visually inspected to check the algorithm’s accuracy: when points defining MT or PA were considered inaccurate, they were manually repositioned. MT was defined as the perpendicular distance between the aponeuroses at the midline of the muscle, whereas PA was defined as the angle between fascicles and the deep aponeurosis (average value of four fascicles) (Seynnes et al. 2015). Fascicle length (FL) was calculated as FL = MT/sen (PA) whereas muscle belly length (BL, the projection of the fascicle on the MTU plane) was calculated as BL = FL cos (PA) (Monte et al. 2022a, b; Wakeling et al. 2011). The longitudinal displacement of the muscle belly was considered to represent the combined elongation of the distal aponeurosis, muscle, and free tendon (i.e., of the GM muscle–tendon unit).

The MTJ_GM_ position was tracked manually (Tracker 6.1.3) to obtain tendon elongation, i.e. the (frame by frame) difference between the MTJ_GM_ position and the MTJ_GM_ position at rest.

All ultrasound data were filtered with a second-order low pass (5 Hz) Butterworth filter.

The ankle moment produced by the plantar-flexor muscles was corrected for the gravitational moment (determined during a passive joint rotation driven by the dynamometer) (Bakenecker et al. 2019); this correction aimed to derive the net ankle torque, which was filtered with a second-order low-pass Butterworth filter (25 Hz).

Muscle tendon unit stiffness (k_M_) and tendon stiffness (k_T_) were determined by analyzing the torque-longitudinal displacement relationships of the GM muscle belly and that of the MTJ_GM_, respectively.

To reconstruct the torque-displacement curves, torque data (acquired at a frequency of 1000 Hz) were sub-sampled at the ultrasound sampling rate (60 Hz). The curves (each trial for each subject) were fitted with a second-order polynomial function forced to go through zero (Hannah and Folland 2015). Only trials with an R^2^ > 0.9 were considered.

Muscle and tendon stiffness were assessed as the slope of the torque-displacement curve in different force intervals: 0–20%, 20–40%, 40–60%, 60–80%, 80–100% of maximum torque (e.g. Maganaris and Paul 2002) (see Figure S2 in supplementary materials).

The values of MTJ_GM_ displacement, MTU_GM_ displacement, peak torque during MVCs and (muscle and tendon) stiffness at the different torque levels/intervals were averaged across the three trials of each subject. The intra-subject variability was particularly elevated in the case of tendon stiffness data since some patients were not able to modulate the increase in force during the MVCs, despite the visual feedback and the verbal instructions. Torque vs. tendon elongation relationships with an R^2^ < 0.8 were disregarded (R^2^ > 0.9 in all other cases). In 3 participants this was the case for all three measurements (either in pre or post training); they were, thus, excluded from tendon stiffness analysis.

### Walking trials

Spatio-temporal parameters (cadence and stride length) were computed based on kinematic data. Cadence was calculated as the inverse of the time between heel strikes of the foot of the dominant leg. The heel strike was identified as the frame in which the anteroposterior velocity of the marker placed on the calcaneus becomes negative (i.e., backward movement of the foot while in contact with the treadmill). Stride length was calculated as the ratio between treadmill speed and cadence. The 3D knee and ankle joint angles were determined, by means of a custom Matlab script, as the angle between two vectors (i.e. thigh and shank for the knee angle, shank and foot for the ankle angle); since the main movement of these joints during locomotion occurs in the sagittal plane, the changes in these angles reflect flexion/extension movements. The average values obtained over twenty strides (ten at the 2nd and ten at the 4th min) of each walking trial were averaged and used in further analysis. The ankle and knee range of motion (ROM) during the stance phase was calculated as the difference between maximal and minimum values (see Figure S3 in supplementary materials).

The EMG activity of VL, BF, TA, and GM was analyzed over ten strides at the 4th minute of exercise. The EMG signal was filtered with a band-pass third-order Butterworth filter at 20–450 Hz and with a band-stop third-order Butterworth filter at 50 Hz. The root mean square (RMS) was computed using a moving window of 25 ms throughout the entire stride and normalized to the subject’s maximum RMS value registered at baseline (all trials and walking speed) as proposed by Carrier et al. 2011). Cumulative activity per distance travelled (CMAPD) was calculated for each muscle, as proposed by Carrier et al. (2011), as the ratio of RMS to walking speed. Total CMAPD (CMAPD_TOT_) was also calculated as the sum of the CMAPD values of all the muscles investigated (Pincheira et al. 2017).

For the metabolic measurements, the resting oxygen uptake was subtracted from the oxygen uptake during exercise to yield the net oxygen uptake (V̇O_2net_ expressed in mlO_2_ min^−1^ kg^−1^). Net energy cost (C_net_) was determined at each speed as the ratio between V̇O_2 net_ and the treadmill speed (e.g. di Prampero 1986), it was expressed in J m^−1^ kg^−1^ by using an energy equivalent (J mlO_2_^−1^) that takes into account the respiratory exchange ratio (Åstrand and Rodahl 1986). The C_net_ vs. speed curve was fitted with a second-order polynomial function to obtain the optimal walking speed (OWS) (the speed at which C_net_ is the lowest) and the optimal energy cost at OWS that are, respectively, the *x* and *y* coordinates of the apex of the fitted function (Bastien et al. 2005).

## Statistical analysis

Data normality was assessed using a Shapiro-Wilks test. Statistical analysis was performed using Jamovi (v2.4.11) and SPSS 23 (IBM Corp., Armonk, NY, USA). Participants’ characteristics were summarized using descriptive statistical methods. Paired t-tests were used to assess the effect of MOS training for: MTJ_GM_ elongation, MTU_GM_ elongation and peak torque during the MVCs.

Two-way repeated measure ANOVAs (with the factors Training and Intervals/Speed) were performed to investigate the effect of MOS training in terms of energy cost, spatiotemporal parameters, knee and ankle ROM, CMAPD, k_M_ and k_T_ at the different speeds/in the different torque intervals. Bonferroni’s correction was used for the post hoc comparisons. Only the main effects of Training are reported. A significance level of 0.05 was used for all statistical tests.

In several cases no interaction effect was observed, but since the main aim of the study was to investigate the pre-post training differences, paired t-tests were used to investigate the effect of training at specific intervals/speeds. In case the normality of the data was not verified, Wilcoxon and Kruskal–Wallis tests were performed.

## Results

In tables and figures, data are reported as means and standard deviations.

### Participants characteristics

Of the 21 patients recruited, 4 were excluded from the final analysis either because they drop out just after pre-test (3) or did not perform the experiments after training (1). In the 17 patients who completed the entire protocol, attendance was 92 ± 9%.

The demographical and physical characteristics of patients (at baseline) are reported in Table 1. Patients’ disease onset ranged from 1 to 20 years, and the values of glycated hemoglobin ranged from 38 to 72 mmol/molHb; all participants were moderately active (an IPAQ score < 700 indicates a sedentary lifestyle and an IPAQ score > 2500 indicates physically active subjects).

**Table 1:** Baseline characteristics of the participants. Data are means ± SD

Age (years)	61.5 ± 5.3
Gender (M/F)	9/8
Body mass (kg)	79.2 ± 11.5
Stature (m)	1.71 ± 0.10
BMI (kg m^−2^)	27.1 ± 2.7
IADL (score)	8.0 ± 0.0
ADL (score)	6.0 ± 0.0
IPAQ (MET/week)	1189 ± 290
Disease duration (years)	7.71 ± 7.52
Glycated haemoglobin (mmol/molHb)	50.9 ± 8.8
Glycated haemoglobin (3yrs)	53.3 ± 8.8
Glucose (mmol/L)	6.94 ± 1.72
Creatinine (μmol/L)	70.1 ± 12.9
Triglycerides (mmol/L)	1.20 ± 0.46
Total cholesterol (mmol/L)	3.81 ± 0.75
HDL Cholesterol (mmol/L)	1.30 ± 0.31

No changes over time were observed during the training sessions for CR10 (1.98 ± 0.98, 1.76 ± 0.80 and 1.46 ± 0.83 average values in the 1st, 5th and 10th week, respectively) and NPRS (2.34 ± 1.22, 1.89 ± 0.70 and 1.74 ± 1.12 in the 1st, 5th and 10th week, respectively); these scores indicate a low perceived exercise intensity and low localized ankle pain.

### Muscle and tendon stiffness

No differences in peak torque were observed after training in the MVCs to determine muscle and tendon stiffness; no changes were also observed in MTU elongation whereas tendon elongation increased after MOS training (see Table 2).

**Table 2:** Muscle–tendon evaluation data. Data of tendon and muscle (MTU) stiffness are reported in Figs. 1 and 2. Only tendon elongation and tendon stiffness in the first torque interval were found to differ pre- and post-training. Data are means ± SD

	Baseline	Post-training	
Peak torque (Nm) §	92.81 ± 39.32	94.15 ± 31.04	
Tendon elongation (mm)	13.20 ± 3.69	15.20 ± 3.09	P = 0.047
Peak torque (Nm) #	86.31 ± 33.52	90.63 ± 30.07	
MTU elongation (mm)	15.20 ± 7.91	16.16 ± 6.64	
Tendon stiffness (0–20%)	6.32 ± 3.49	4.20 ± 3.12	P = 0.026

^§^in the experiments to determine tendon stiffness and # in the experiments to determine muscle stiffness

**Fig. 1:**
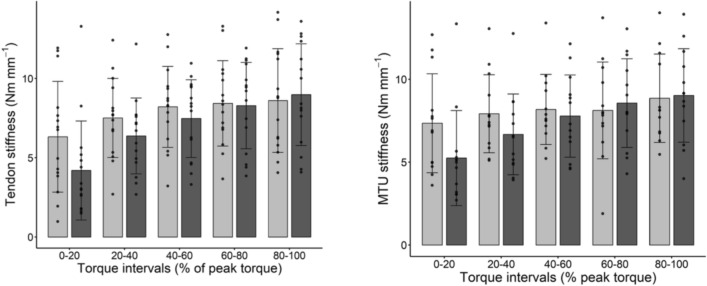
Tendon stiffness (panel on the left) and muscle (MTU) stiffness (panel on the right) in different torque intervals (in % of maximum torque, relative values); light gray bars and dark gray bars indicate pre and post training values, respectively. Data are means and bars represent SD, individual values are reported as well

**Fig. 2:**
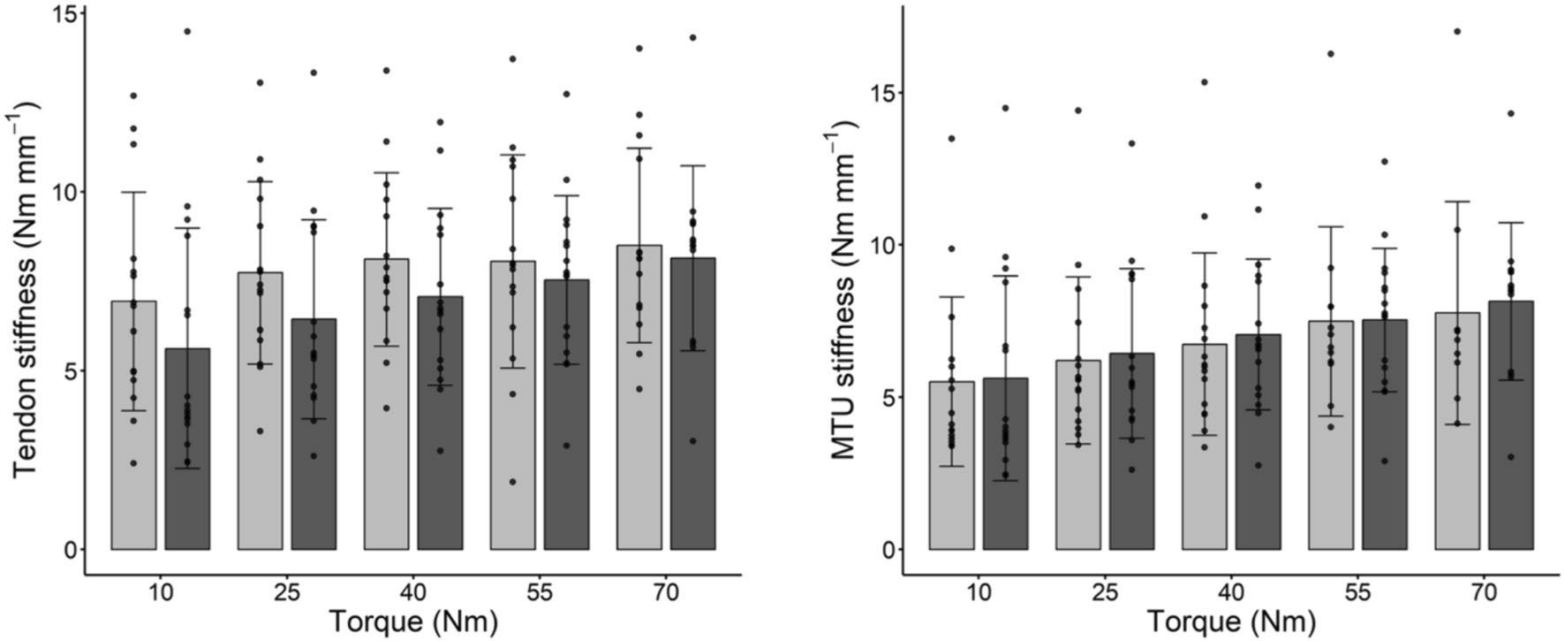
Tendon stiffness (panel on the left) and muscle (MTU) stiffness (panel on the right) at specific torque levels (absolute values, Nm); light gray bars and dark gray bars indicate pre and post training values, respectively. Data are means and bars represent SD, individual values are reported as well

Figures 1 and 2 show the values of tendon (k_T_) and MTU (k_M_) stiffness, expressed in relative and absolute terms, respectively. The main effect of training on k_T_ and k_M_ indicated no significant differences both in the absolute (ANOVA main factor: P = 0.467 and P = 0.736, respectively) and relative values (ANOVA main factor: P = 0.205 and P = 0.485, respectively). No interaction effect between training and intensity was observed in k_T_ and k_M_ both in the absolute (k_T_: P = 0.035; k_M_: P = 0.347) and relative values (k_T_: P = 0.213; k_M_: P = 0.343). However, the values of tendon stiffness tended to be lower after training in the lower range of torque values (0–20% MVC); a paired student t-test in these specific conditions indicates a significant decrease in k_T_ after training (see Table 2).

### Walking trials

**Fig. 3:**
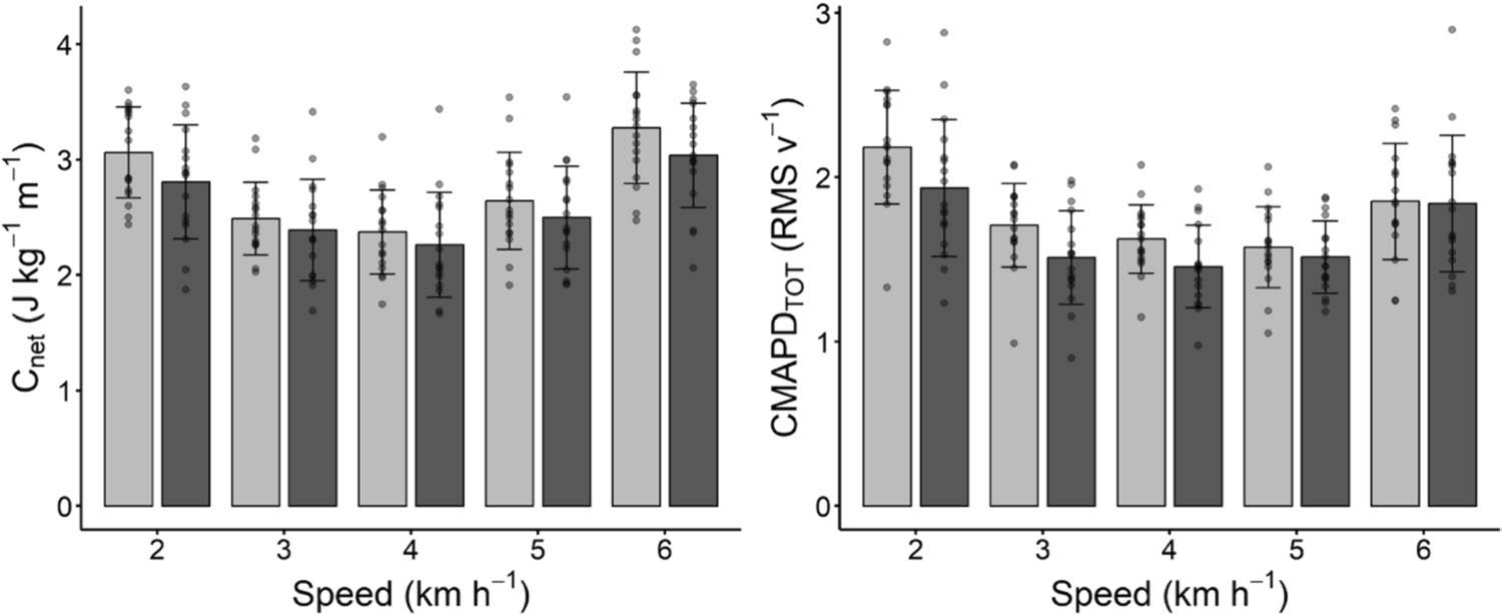
Net energy cost of walking (C_net_, panel on the left) and cumulative activity per distance travelled (CMAPD_TOT_, panel on the right) at the investigated walking speeds; light gray bars and dark gray bars indicate pre and post training values, respectively. Data are means and bars represent SD, individual values are reported as well

The net energy cost of walking at the investigated speeds is reported in Fig. 3 (panel on the left). A reduction in C_net_ was observed after training (ANOVA main factor: P = 0.014) but with no interaction effect between training and speed (P = 0.160). Paired student t-tests indicated a decrease in the energy cost after training at the optimal speed (C_net_ at OWS) as well as at 2 and 6 kmh^−1^ (see Table 3). No changes were observed in the OWS (pre: 3.82 ± 0.32 kmh^−1^; post 3.71 ± 0.76 kmh^−1^, P = 0.538).

**Table 3:** Statistically significant pre-post differences (paired Student’s t test) in MOS training group during the walking trials

	PRE	POST	
C_net_ at OWS (J m^−1^ kg^−1^)	2.34 ± 0.37	2.15 ± 0.34	P = 0.007
C_net_ at 2 kmh^−1^ (J m^−1^ kg^−1^)	3.06 ± 0.39	2.81 ± 0.49	P = 0.014
C_net_ at 6 kmh^−1^ (J m^−1^ kg^−1^)	3.28 ± 0.48	3.04 ± 0.45	P = 0.007
CMAPD_TOT_ at 3 kmh^−1^	1.71 ± 0.25	1.51 ± 0.29	P = 0.025
Ankle ROM at 2 kmh^−1^	17.87 ± 3.09	18.80 ± 3.33	P = 0.037
Ankle ROM at 3 kmh^−1^	20.08 ± 3.18	20.96 ± 3.32	P = 0.042
SL at 2 kmh^−1^ (m)	0.80 ± 0.08	0.83 ± 0.07	P = 0.004
SL at 3 kmh^−1^ (m)	1.02 ± 0.08	1.05 ± 0.09	P = 0.015
SL at 4 kmh^−1^ (m)	1.24 ± 0.08	1.26 ± 0.09	P = 0.025
SF at 2 kmh^−1^ (Hz)	0.70 ± 0.08	0.67 ± 0.06	P = 0.003
SF at 3 kmh^−1^ (Hz)	0.82 ± 0.06	0.80 ± 0.06	P = 0.015
SF at 4 kmh^−1^ (Hz)	0.90 ± 0.06	0.89 ± 0.06	P = 0.046

*C*_net_ net energy cost of walking, *OWS* optimal walking speed, *CMAPD*_TOT_ total cumulative activity per distance travelled, *ROM* range of motion, *SL* stride length, *SF* stride frequency

No changes in CMAPD_GM_ and CMAPD_TOT_ (Fig. 3, panel on the right) were observed after training (ANOVA main factor: P = 0.084 and P = 0.080, respectively) and no significant interaction between training and speed was observed (P = 0.475 and P = 0.340, respectively). A paired student t-test indicated a decrease in CMAPD_TOT_ after training at 3 kmh^−1^ (see Table 3).

**Fig. 4:**
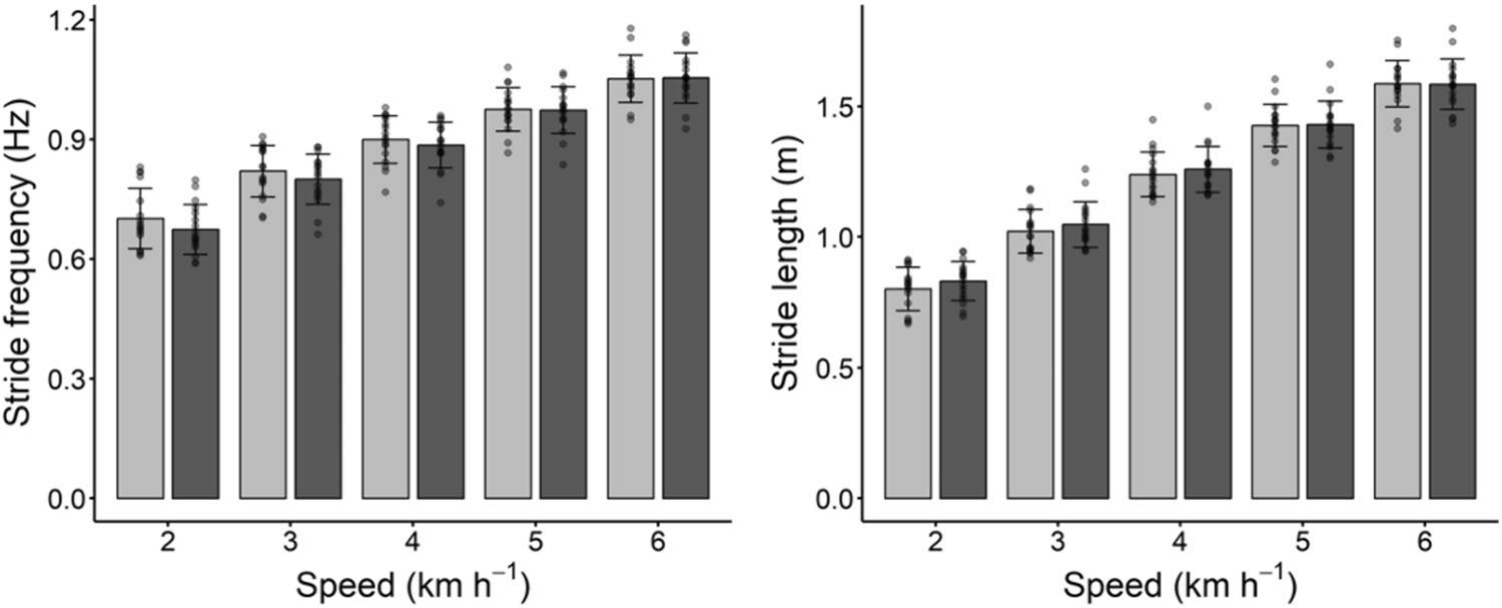
Stride frequency (panel on the left) and stride length (panel on the right) at the investigated walking speeds; light gray bars and dark gray bars indicate pre and post training values, respectively. Data are means and bars represent SD, individual values are reported as well

Differences in spatiotemporal parameters (Fig. 4) were observed after training (ANOVA main factor: P < 0.001 for both cadence and stride length), with no significant interaction between training and speed (stride frequency: P = 0.120; stride length: P = 0.100). Paired student t tests indicate a decrease in cadence and an increase in stride length after training at 2, 3 and 4 kmh^−1^ (see Table 3).

**Fig. 5:**
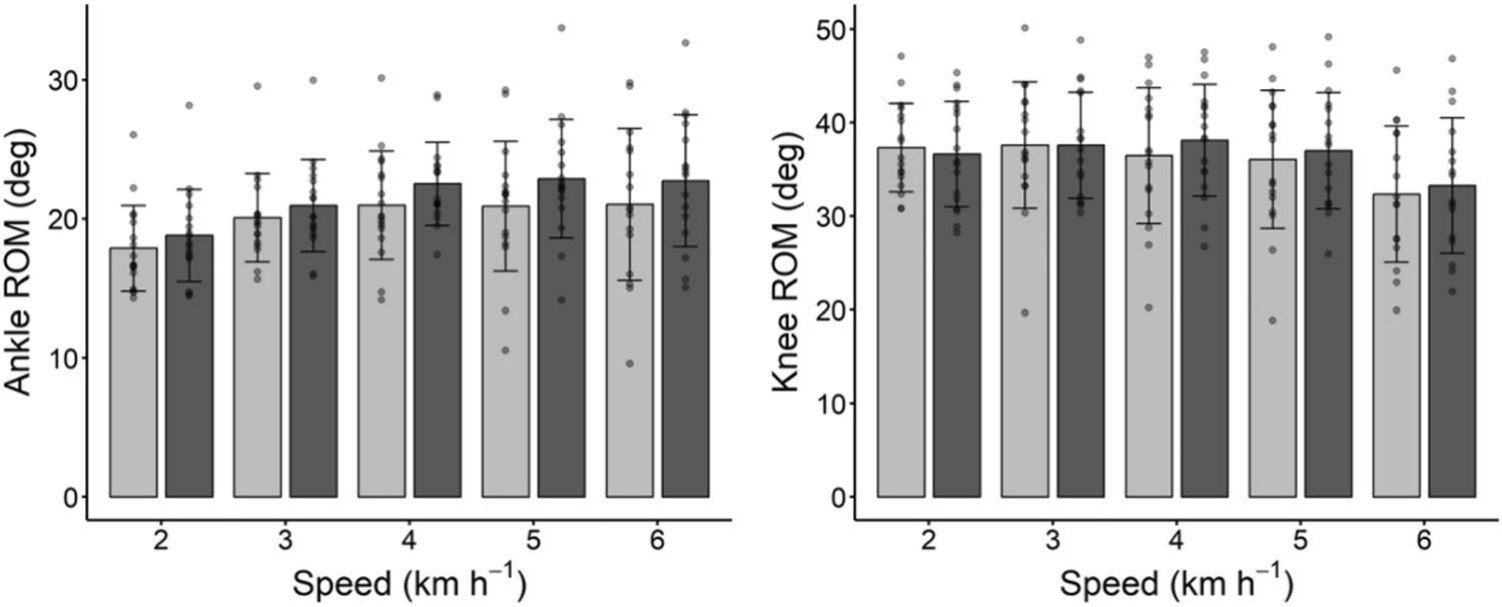
Ankle (panel on the left) and knee (panel on the right) ROM at the investigated walking speeds; light gray bars and dark gray bars indicate pre and post training values, respectively. Data are means and bars represent SD, individual values are reported as well

A significant difference was observed in ankle ROM after training (ANOVA main factor: P = 0.043), while this was not the case for knee ROM (P = 0.762). No interaction effect was observed in knee and ankle ROM (P = 0.973 and P = 0.706, respectively) (see Fig. 5). However, the range of motion of the ankle tended to increase after training; a paired student t-test indicates a significant increase at 2 and 3 kmh^−1^ (see Table 3). The increased ankle ROM (1.40 ± 0.49°, on average, at all walking speeds) was mainly driven by increased dorsiflexion (1.31 ± 0.43°).

## Discussion

This study aimed to determine the effects of a ten-week MOS training protocol on muscle and tendon stiffness, and on the walking capability of diabetic patients.

Our data showed an increase in Achilles tendon elongation after MOS training, without appreciable changes in maximal torque nor in MTU elongation. In addition, even if no changes were observed in muscle stiffness, a decrease in tendon stiffness was observed in the crimped region (0–20% of MVC), suggesting an improvement in tendon elastic capacity.

These training adaptations have a transfer into the locomotor capabilities of these patients. Indeed, during the post-training walking tests (at the slowest tested speeds) we observed an increase in the ankle ROM, an increase in stride length, a decrease in cadence, and a decrease in the energy cost of walking. These changes in “walking capability” are coherent with the changes that could be expected after passive stretching of the plantar-flexor muscles, and this suggests that this form of training could induce a positive adaptation for this population.

### Muscle and tendon stiffness

The Achilles tendon plays a crucial role in transmitting muscle force from the active components of the plantar-flexor muscles to the skeleton. For this reason, its mechanical adaptation (i.e., stiffness) in response to ageing, training and disease is well investigated in the scientific literature. Achilles tendon stiffness typically decreases as a function of age due to tendon deterioration (e.g., lower collagen turnover and packing density; Naresh and Brodsky 1992), increased collagen molecules crimp angle (Patterson-Kane et al. 2012) and/or decreased water content (Ippolito et al. 1980). On the other hand, tendon stiffness could increase in response to high values of AGEs, which increase the non-enzymatic cross-linking of the soft tissues (Reddy 2004). Indeed, diabetic patients are characterized by higher values of Achilles tendon stiffness compared to age-matched healthy controls (Couppé et al. 2016; Petrovic et al. 2018).

Our data indicate that 10 weeks of MOS training were able to improve maximum tendon elongation and to reduce tendon stiffness in the first region of the force–elongation curve (0–20% of Fmax). These adaptations could be functionally relevant since (i) the operating length of the Achilles tendon during walking is within 50% of the maximum Achilles tendon force (Finni et al. 2001; Maganaris and Paul 2002; Monte et al. 2022a, b); and (ii) for a given (maximum) force/torque, an increase in maximum tendon elongation represents an increase in the maximum elastic energy capacity of the Achilles tendon. Given that AGEs in diabetic patients contribute to collagen disorganization in the tendons (Guney et al. 2015; Suzuki et al. 2022) and to a reduction in fiber sliding (Li et al. 2013), we can hypothesize that MOS stretching leads to improved orientation and sliding of tendon collagen fibres (Franchi et al. 2007) thereby enhancing overall tendon elongation. Therefore, our data suggest that 10 weeks of MOS training could improve the Achilles tendon mechanical capacity in people with diabetes, with a possible positive transfer in their functional capacity (e.g., walking).

MTU stiffness is another functionally relevant parameter due to its association with the rate of force development and the risk of falls (Perry et al. 2007). Although the adverse effects of diabetes on muscle/MTU mechanics and function are still unclear, our data indicate no significant effect of MOS training on muscle/MTU elongation or stiffness in people with T2D. As measured in this study (based on values of muscle-belly displacement), MTU stiffness evaluation takes into account also the elongation of the tendon, aponeuroses and the intramuscular connective tissues of the investigated muscle. As reported by Herzog (2019), the tendon and the aponeuroses of a given MTU are anatomically but not mechanically in-series and, thus, it is possible to assume that MOS training has a reduced effect at the “muscle” level, due to a possible “damper effect” of the tendon. This speculation could also partially explain the results obtained by Ikeda et al. (2019, 2021), where MOS training did not have any effect on muscle force capacity (as is the case in our study).

### Walking trials

Spatiotemporal parameters of walking have been investigated in several contexts due to their association with disability and functional capacity. For example, step length decreases as a function of age and is reduced in people with Parkinson’s disease (Nardello et al. 2017), diabetes (Martinelli et al. 2013; Petrovic et al. 2016) and other pathological conditions (see Perry (1992) for a revision about the topic); for a given velocity, walking with a longer stride length means a decrease in walking cadence.

In accordance with other studies (e.g. Park et al. 2019; Thong-On et al. 2019; Watt et al. 2011), we observed an increase in stride length and a decrease in cadence following a stretch training protocol focused on the ankle joint/the Achilles tendon function. We also observed an increase in the ankle range of motion during walking (albeit significant only at the lowest tested speed), mainly driven by an increase in dorsiflexion. A positive correlation between the ankle dorsiflexion range of motion and the stride length was reported in diabetic patients with peripheral neuropathies (e.g. Martinelli et al. 2013). Accordingly, Petrovic et al. (2018) suggest that shorter steps in T2D patients relate to reduced ankle range of movement, reduced tendon elongation and increased tendon stiffness (as observed in this study). A training modality able to promote mobility at the ankle level (such as the one proposed in this study) is, thus, expected to result in an improvement in the quality of gait spatiotemporal parameters.

Changes in the walking pattern in T2D patients have been suggested to be responsible for their higher energy expenditure during walking, especially in the case of peripheral neuropathies (Petrovic et al. 2016; Caron et al. 2018). The larger energy expenditure in T2D patients was also attributed to their increased tendon stiffness, which would reduce the elastic energy stored during walking thus requiring a relatively greater contribution from the plantar flexor muscles (e.g. Petrovic et al. 2018). Data reported in this study indicate that energy cost could be reduced after stretch training, and MOS training effectiveness is expected to be even more effective in patients with peripheral neuropathies.

### Participants

We recruited moderately active participants (IPAQ: 1189 ± 290 MET/week) with fairly well controlled diabetes (HbA1c: 50.9 ± 8.8 mmol/molHb).

Our training protocol had a very high compliance (92 ± 9%), whereas other training methods (i.e. aerobic training, high-intensity interval training, and resistance training) are less tolerated by this population, with an adherence that is often lower than 80% (Bullard et al. 2019; MacDonald et al. 2021). The high participation may be attributed to the low levels of perceived exertion and pain reported by the subjects during training. The participants were supervised regarding the oscillatory frequency and the body asset (e.g. they had to maintain the knee angle extended and the rubber band properly held and positioned), but the stretch intensity (e.g. the range of motion) was self-selected. The training protocol was designed to stretch the ankle almost maximally but the low CR10 and NPRS scores indicate that participants tended to be “conservative”; this suggests that training effectiveness could be higher when also controlling the ROM during each stretching session.

Our results indicate that MOS training could reduce the energy cost of walking in patients with a moderate physical active lifestyle. To note, many diabetic patients are inactive or insufficiently active (Dai et al. 2023); thus, MOS training effectiveness is expected to be higher in less active patients.

Regarding glycaemic control, the American Diabetes Association (American Diabetes Association 2021) indicates a threshold of 53 mmol/molHb for a substantially increased risk of adverse effects of diabetes. In our study only 6 out of 17 participants had HbA1c levels larger than that; thus, MOS training effectiveness is expected to be higher in patients with a lower glycaemic control (e.g. higher HbA1c levels).

## Limitations

In this study, we did not test a group of T2D patients as controls. Although this could be a limitation, a control group is generally necessary when some confounding factors are expected (e.g. changes in tendon stiffness or walking capabilities) in the absence of the intervention. Since T2D is a chronic disorder with limited short-term complications, we did not expect a significant influence of possible confounding factors in 10 weeks. However, future studies could consider recruiting a control group to better elucidate the effect of a stretching training protocol on people with T2D.

Another possible limitation was that the study was not blinded. The examiners were the same before and after training, to ensure repeatable conditions of testing and to avoid the occurrence of possible confounding factors due to inter-operator variability. In addition, data were analyzed in aggregate form, using Matlab scripts.

Most of the participants had limited or no experience of walking on a treadmill; even if a familiarization period was proposed to each participant, the decrease in energy cost observed after training could be partially attributed to habituation. Walking on the level could be a better choice when testing these patients/elderly people.

The tendon stiffness data of two subjects were excluded from the analysis for their inability to correctly perform the assigned task (a constant rise in force up to maximal values) even if we utilized real-time feedback to control the quality of data during muscle–tendon evaluation. Thus, with these patients it is suggested, to perform several familiarization sessions.

To evaluate training efficacy, the participants were tested the week after the conclusion of MOS protocol. However, the long-term impact of this stretching modality was not examined via a retention test; hence, this aspect needs investigation in future studies.

## Conclusion

This study indicates the effectiveness of 10 weeks of MOS training in reducing tendon stiffness and the energy cost during walking in moderately physically active diabetic patients. The effects of this training method were quite limited because the training intensity was probably too low (self-selected by the subjects). However, this training protocol requires no specific instrumentation, can be easily performed at home, and has a high adherence. Thus, this simple stretching protocol could be useful to mitigate mechanical tendon deterioration and physical inactivity related to diabetes, thereby extending daily movement duration.

## Supplementary Information

Below is the link to the electronic supplementary material.

Supplementary file1 (DOCX 24330 KB)
