# Supplementary material for: Effects of minute oscillation stretching training on muscle and tendon stiffness and walking capability in people with type 2 diabetes
Source: Eur J Appl Physiol. 2024 Sep 9;125(1):183–95. doi: 10.1007/s00421-024-05596-y (PMC11746953; doi:10.1007/s00421-024-05596-y)
Supplement: Supplementary file 1 — Supplementary file1 (DOCX 24330 KB) [file 421_2024_5596_MOESM1_ESM.docx]

**SUPPLEMENTARY MATERIALS**

**Effects of minute oscillation stretching training on muscle and tendon stiffness and walking capability in people with type 2 diabetes**

Riccardo Magris^1^, Andrea Monte^1^, Francesca Nardello^1^, Michele Trinchi^1^, Nicolò Vigolo^2^, Carlo Negri^3^, Paolo Moghetti^2^, Paola Zamparo^1^

**MATERIALS AND METHODS**

^
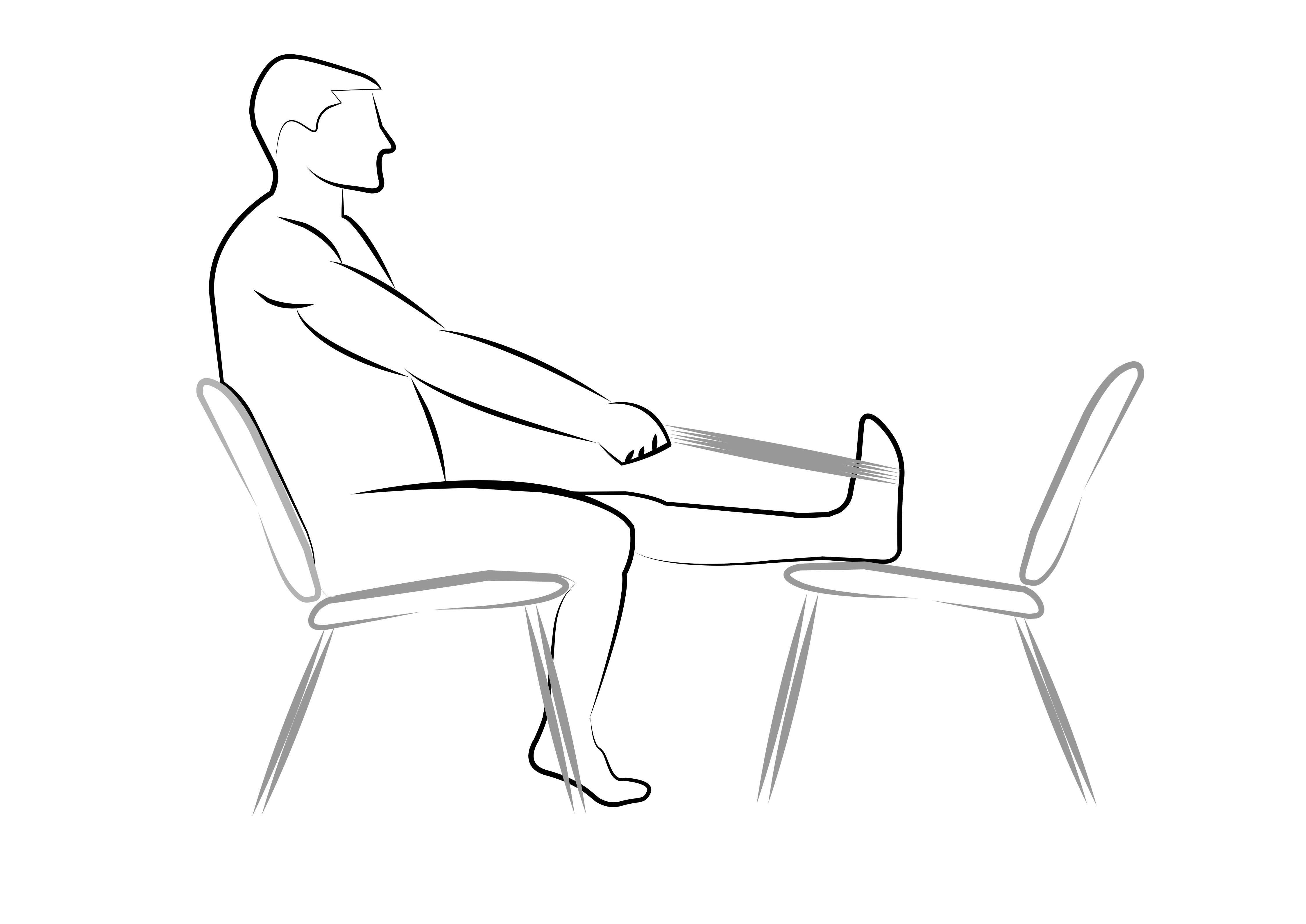
^

**Figure S1.** During training, the participants were requested to sit on a chair and to set their foot over another chair, positioned in front of them. By stretching a rubber band passed under the sole of the feet they “passively” reached maximum ankle dorsiflexion; by releasing the rubber band the ankle joint returned to normal passive plantarflexion (see text for details).


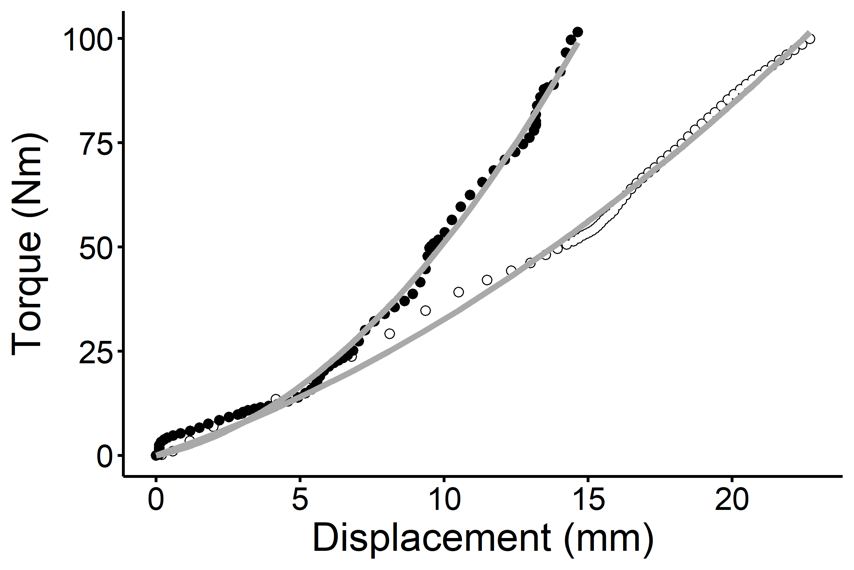


**Figure S2.** Torque vs. displacement relationship in the Achilles tendon (circles) and in the GM muscle tendon unit (squares) in a representative subject. Tendon stiffness and muscle stiffness were calculated as: *i*) the slope of the torque-displacement curve in different force intervals: 0-20%, 20-40%, 40-60%, 60-80%, 80-100% of maximum torque and *ii*) the slope of the torque-displacement curve at specific torque levels: 10, 25, 40, 55, 70 Nm.


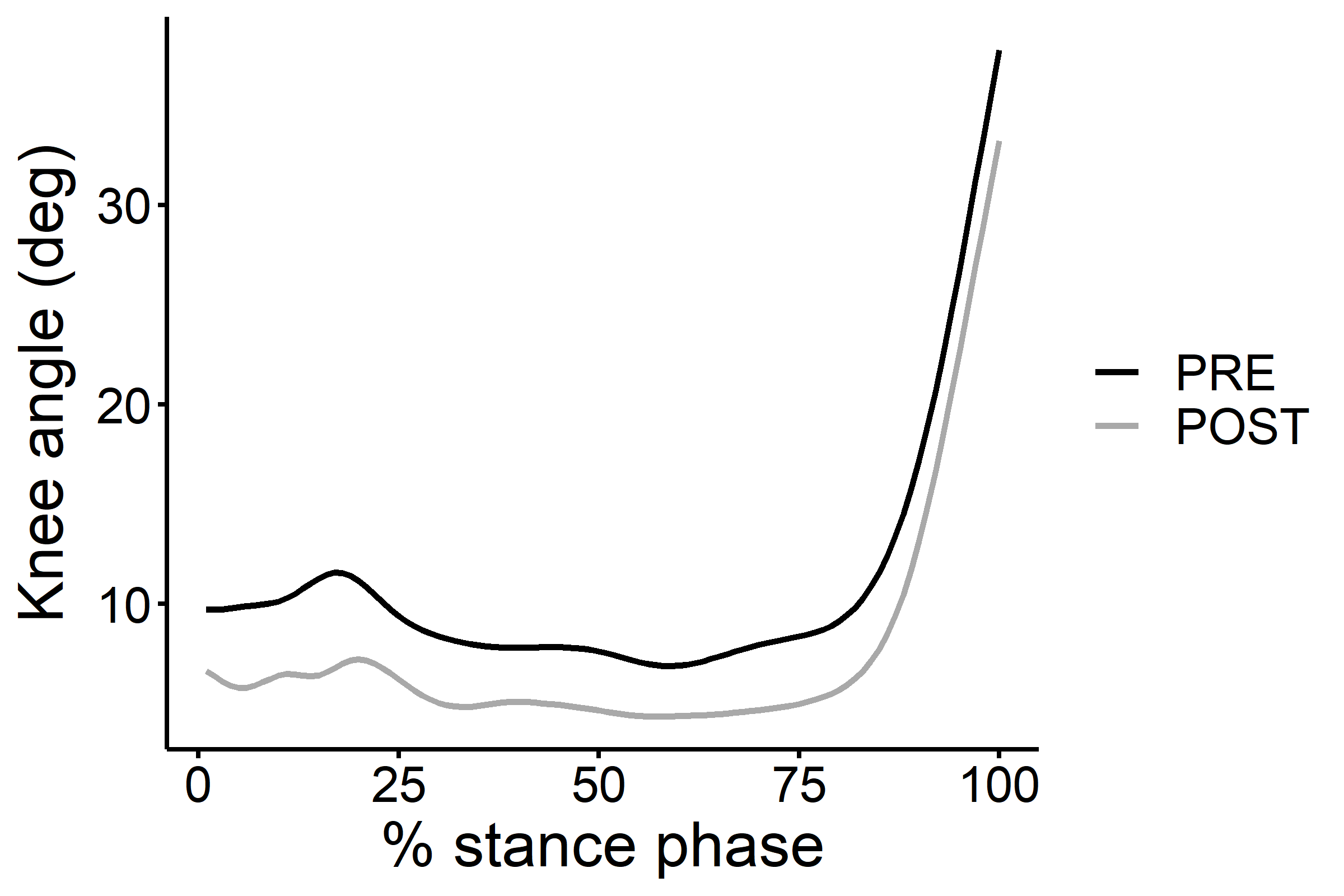

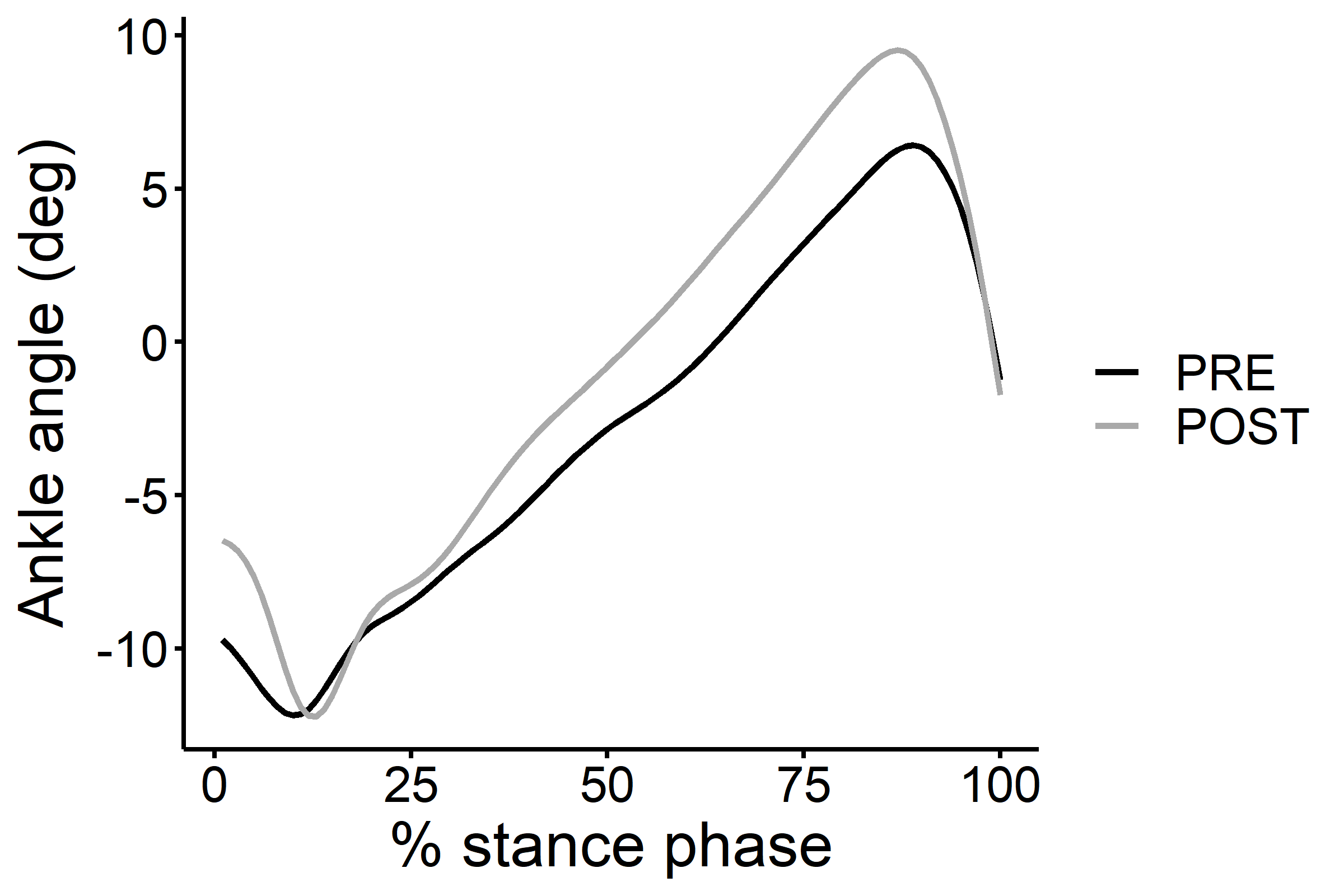


**Figure S3.** Ankle (panel on the left) and knee (panel on the right) angles during the stance phase (pre training values in gray and post training values in black) in a representative subject.
